# Efficacy of visceral fat estimation by dual bioelectrical impedance analysis in detecting cardiovascular risk factors in patients with type 2 diabetes

**DOI:** 10.1186/s12933-019-0941-y

**Published:** 2019-10-22

**Authors:** Yoko Omura-Ohata, Cheol Son, Hisashi Makino, Ryo Koezuka, Mayu Tochiya, Tamiko Tamanaha, Ichiro Kishimoto, Kiminori Hosoda

**Affiliations:** Division of Diabetes and Lipid Metabolism, National Cerebral and Cardiovascular Center, 6-1 kishibeshin-machi, Suita, Osaka 564-8565 Japan

**Keywords:** Visceral fat, Type 2 diabetes, Impedance, Cardiovascular risk factor

## Abstract

**Background:**

Visceral fat area (VFA) is a good surrogate marker of obesity-related disorders, such as hypertension, dyslipidemia and glucose intolerance. Although estimating the VFA by X-ray computed tomography (CT) is the primary index for visceral obesity, it is expensive and requires invasive radiation exposure. Dual bioelectrical impedance analysis (BIA) is a simple and reliable method to estimate VFA; however, the clinical usefulness of dual BIA remains unclear in patients with type 2 diabetes (T2D).

**Methods:**

We estimated the VFAs by dual BIA and CT in 98 patients with T2D and assessed anthropometric parameters, blood test results, and the presence of comorbid hypertension and dyslipidemia. We compared the correlation between the VFAs examined by dual BIA and CT. Furthermore, we performed the receiver operating characteristic (ROC) analyses for the VFAs to detect the presence of comorbid hypertension and/or dyslipidemia with T2D, which are major comorbidities of visceral obesity, and estimated the area under the curve (AUC).

**Results:**

The measurement error between the VFAs by dual BIA and CT was significantly higher among patients with brain natriuretic peptide (BNP) ≥ 100 pg/mL than those with BNP < 100 pg/mL (39.2% ± 31.1% vs. 24.1% ± 18.6%, P < 0.05). After excluding patients with BNP ≥ 100 pg/mL, the VFA by dual BIA significantly correlated with the VFA by CT (r = 0.917; P < 0.0001). The AUC in the ROC analysis for the VFA by dual BIA to detect the presence of comorbid hypertension and/or dyslipidemia with T2D was almost equivalent to that for the VFA by CT.

**Conclusions:**

In patients with T2D without elevated BNP > 100 pg/mL as indicator for fluid accumulation interfering with BIA, estimation of the VFA by dual BIA significantly correlated with that by CT and also detected comorbid hypertension and/or dyslipidemia with T2D equivalent to those detected by CT. Hence, dual BIA could be an alternative to CT as a standard method for estimating the VFA in patients with diabetes.

## Background

Obesity is an established risk factor for metabolic and cardiovascular diseases [1–4] and is defined as excessive lipid accumulation in the adipose tissue [5]. The adequate distribution of the adipose tissue is imperative because it reflects different pathophysiology [6]. Previous studies on the morbidity of obesity have indicated that obesity-related diseases are more associated with visceral fat rather than the accumulation of whole body fat [7], especially in Asian countries including Japan [8]. Although precisely measuring entire visceral fat amount is difficult, the visceral fat area (VFA) estimated by X-ray computed tomography (CT) at umbilical level has been reported as a good surrogate marker of obesity-related disorders, especially such as hypertension, dyslipidemia and glucose intolerance [9–12]. However, this method is expensive and requires radiation exposure, and thus, it is not useful for ubiquitous and frequent use. In contrast, dual bioelectrical impedance analysis (BIA), which measures the bioelectrical impedance of the entire abdomen and its surface with a dual current path, is a simple and reliable method to estimate visceral fat accumulation [13]. In fact, it is considered better than the conventional BIA using only one current path. A significant correlation between the VFA measured by dual BIA (VFA-BIA) and the VFA measured by CT (VFA-CT) has been reported in healthy subjects [14]. However, correlation between the VFA-BIA and the VFA-CT has not reported in patients with type 2 diabetes (T2D). Thus, this study aimed to assess the correlation between the VFA-BIA and VFA-CT in patients with T2D and examined the clinical usefulness of the VFA-BIA to evaluate visceral obesity by comparing to the VFA-CT the ability of detecting the presence of comorbid hypertension and/or dyslipidemia with T2D patients.

## Methods

### Study subjects

In this study, we enrolled consecutive 98 (73 males and 25 females) patients with T2D who were admitted to the Department of Endocrinology and Metabolism at the National Cerebral and Cardiovascular Center for glucose control and had examination of both VFA-CT and VFA-BIA on the same day between October 2011 and September 2012. Patients with distinct edema, symptomatic heart failure (New York Heart Association class II–IV), nephrotic syndrome, and pacemaker implantation were excluded from the analysis. The physical examination of patients included the height, body weight, waist circumference (WC), and blood pressure measurements. The WC was measured at the umbilical level in the late exhalation phase while standing. Hip circumference was measured around the widest portion of the buttocks. Blood pressure was measured once with mercury sphygmomanometer after patients were quietly seated on admission. The body mass index (BMI) was calculated as the body weight (kg) divided by the square of height (m^2^). In addition, the waist-to-hip ratio (WHR) was calculated as the WC divided by the hip circumference. Patients’ medical histories were obtained from medical records, including a history of heart failure, renal failure, diabetic nephropathy, and the existence of cardiomegaly as well as the current use of diuretics and oral hypoglycemic agents. Hypertension was defined as; SBP ≥ 140 mmHg and/or DBP ≥ 90 mmHg or under antihypertensive treatment. Dyslipidemia was defined as follows; fasting triglycerides ≥ 150 mg/dL and/or HDL-C < 40 mg/dL or receiving lipid-lowering drugs.

In the statistical analyses, we divided the patients into two groups by BNP levels. We used 100 pg/mL as the cutoff value because BNP threshold of 100 pg/mL is proposed for suspected heart failure by several papers including NICE Guideline No 5 by National Clinical Guideline Centre (UK) [15–17].

### Laboratory methods

Blood samples were drawn from patients after a 12-h overnight fast. The plasma glucose concentration was measured by the glucose oxidase method, and serum concentrations of insulin and C-peptide were assayed using double-antibody radioimmunoassay. In addition, serum total cholesterol and triglyceride concentrations were determined using enzymatic methods. Following heparin and calcium precipitation, high-density lipoprotein cholesterol (HDL-C) was measured by an enzymatic method. Furthermore, dyslipidemia [hypertriglyceridemia (fasting triglycerides ≥ 150 mg/dL) and/or low HDL cholesterolemia (HDL-C < 40 mg/dL) or under treatment] and hypertension [systolic blood pressure (SBP) ≥ 140 mmHg and/or diastolic blood pressure (DBP) ≥ 90 mmHg or under treatment] were assessed as obesity-related cardiovascular risk factors. A homeostasis model assessment for insulin resistance (HOMA-IR) was calculated to assess the insulin resistance, using the formula [fasting plasma insulin (μU/mL) × fasting glucose (mg/dL)]/405 [18].

### Measurement of VFA

We estimated the VFA by both CT and dual BIA on the same day. After an overnight fast and urination, VFA was estimated by impedance using dual BIA (HDS 2000; Omron Co. Ltd., Kyoto, Japan). Then, CT was performed before lunch using the multislice device (Toshiba Aquilion ONE; Toshiba Co. Ltd., Tokyo, Japan). We obtained a single axial tomographic slice at the L4–L5 level using 120 mV and 385 mA, and the VFA at the umbilical level was semi-automatically determined using the image analysis software Fat Scan Premium Version 5.0 (East Japan Institute of Technology Co., Ltd, Ibaraki, Japan) by two independent researchers.

### Dual BIA instrument

Dual BIA instrument calculates the cross‐sectional area of intra‐abdominal fat at the level of umbilicus based on the measurement of electrical potentials resulting from applying small electrical currents in two different body space (Omron Healthcare Co., Ltd., Kyoto, Japan). The underlying principle of this determination has been previously described in detail by several studies including ours [13, 14, 19]. Briefly, the dual-BIA measures impedance reflecting the FFV and the SFV by passing current via respective pathways. The two sets of electrodes are for limb and truncal placement. The four electrodes are placed on hands and legs and measure impedance in an axial direction of the abdomen reflecting FFV. The four pairs of truncal electrodes are placed on the abdominal and dorsal regions and measure abdominal surface impedance reflecting SFA. Using the VFA measured by X-ray CT as a reference, an algorithm was constructed to calculate the VFA using 1/Zt, Zs, reflecting FFV and SFV respectively and A, B reflecting the abdominal shape.

VFA-BIA was calculated as follows:

$${\text{VFA-BIA}} =\upalpha1{\text{A}} +\upalpha2{\text{B}}^{2} -\upalpha3\left( {{\text{A}}^{2} + {\text{B}}^{2} } \right)^{{{1 \mathord{\left/ {\vphantom {1 2}} \right. \kern-0pt} 2}}} {\text{Zs}} -\upalpha4/{\text{Zt}} +\upvarepsilon$$

where A is the abdominal anteroposterior diameter, B is the abdominal transverse diameter, Zs is surface impedance, Zt is truncal impedance, and α1-α4, ε are the constants determined by validation study.

### Statistical analysis

The correlation between the VFA-BIA and the VFA-CT was determined using Pearson’s correlation coefficient. The multivariate regression analysis was performed to detect independent association between measurement error and following factors; age, gender, BMI, diabetes duration, HbA1c, blood sugar, eGFR, BNP and history of heart failure. Bland–Altman plot was conducted to assess the agreement between VFA-CT and VFA-BIA. The sensitivity and specificity of the VFA measurement at the diagnosis of hypertension (SBP ≥ 140 mmHg and/or DBP ≥ 90 mmHg or under treatment) and/or dyslipidemia (HDL-C < 40 mg/dL and/or triglycerides ≥ 150 mg/dL or under treatment) were calculated using the receiver operating characteristic (ROC) analyses. The diagnostic ability of each test was compared by calculating the area under the curve (AUC). Optimal cutoff value was determined by Youden index method [20]. Statistical tests for the comparison of AUCs were conducted by the nonparametric approach proposed by Delong et al. [21]. Two-tailed P < 0.05 was considered statistically significant. All data were expressed as mean ± standard deviation. Statistical analyses were conducted using JMP ver.8.0 (SAS Institute Inc., Cary, NC, USA).

## Results

### Patient characteristics

Table 1 shows the clinical, anthropometric, and metabolic characteristics of patients. In this study, we enrolled 98 patients (73 males and 25 females; mean age: 66.2 ± 4.0 years; range: 22–84 years). The mean BMI of patients was 25.0 ± 4.0 kg/m^2^. The mean WC of patients was 89.3 ± 12.4 cm (males: 90.9 ± 13.0 cm; females: 84.1 ± 8.6 cm), and the mean WHR was 0.94 ± 0.08. The VFA-CT and VFA-BIA were 116.1 ± 65.6 cm^2^ and 83.7 ± 46.0 cm^2^, respectively. The mean duration of diabetes was 15.5 ± 11.8 years, and mean HbA1c and fasting blood sugar levels were 8.9% ± 1.9% (73.5 ± 20.7 mmol/mol) and 156.9 ± 57.3 mg/dL, respectively. Furthermore, the mean C-peptide level was 2.7 ± 1.8 ng/mL.

**Table 1:** Patient characteristics

	All patients (*N* = 98)	Males (*N* = 73)	Females (*N* = 25)
N	98	73	25
Age, years	66.2 ± 10.9	66.2 ± 11.0	66.9 ± 11.5
BMI, kg/m^2^	25.0 ± 4.0	25.5 ± 4.3	23.6 ± 3.0
WC, cm	89.3 ± 12.4	90.9 ± 13.0	84.1 ± 8.6
WHR	0.94 ± 0.08	0.96 ± 0.08	0.91 ± 0.07
Duration of diabetes, years	15.5 ± 11.8	15.9 ± 12.2	14.2 ± 10.2
HbA1c, % (mmol/mol)	8.9 ± 1.9 (73.5 ± 20.7)	9.0 ± 2.0 (74.5 ± 22.3)	8.6 ± 1.4 (70.4 ± 15.3)
FBS, mg/dL	156.9 ± 57.3	157.7 ± 62.1	154.9 ± 41.4
SBP, mmHg	132.1 ± 21.9	132.1 ± 20.6	132.2 ± 25.8
DBP, mmHg	72.1 ± 14.7	71.3 ± 14.2	74.3 ± 16.1
Total cholesterol, mg/dL	181.9 ± 39.4	179.2 ± 39.5	189.5 ± 39.2
HDL-cholesterol, mg/dL	43.8 ± 13.5	41.7 ± 13.2	49.7 ± 12.9
LDL-cholesterol, mg/dL	105.3 ± 31.7	104.4 ± 31.5	108.0 ± 32.9
Triglyceride, mg/dL	175.7 ± 107.5	181.7 ± 111.6	157.3 ± 94.0
IRI, IU/mL	8.9 ± 8.3	9.6 ± 9.2	6.8 ± 4.5
HOMA-IR	3.7 ± 4.3	4.1 ± 4.7	2.8 ± 2.5
BNP, pg/mL	57.7 ± 78.9	51.3 ± 64.7	82.6 ± 120.7
eGFR, mL/min/1.73 m^2^	61.1 ± 24.2	60.2 ± 25.2	63.6 ± 20.6
VFA-CT, cm^2^	116.1 ± 65.6	125.6 ± 70.9	88.4 ± 35.5
VFA-BIA, cm^2^	83.7 ± 46.0	91.0 ± 48.8	62.6 ± 27.9
Diabetes mellitus, N (%)	98 (100.0)	73 (100.0)	25 (100.0)
Hypertension, N (%)	72 (73.5)	55 (75.3)	17 (68.0)
Dyslipidemia, N (%)	88 (89.8%)	66 (90.4%)	22 (88.0%)

Data are presented as mean ± standard deviation

*N* number, *BMI* body mass index, *WC* waist circumference, *WHR* waist/hip ratio, *HbA1c* glycated hemoglobin, *FBS* fasting blood sugar, *HDL* high-density lipoprotein, *LDL* low-density lipoprotein, *IRI* immunoreactive insulin, *HOMA-IR* homeostasis model assessment for insulin resistance, *eGFR* estimated glomerular filtration rate, *VFA* visceral fat area, *CT* computed tomography, *BIA* bioelectric impedance analysis

### Measurement error between the VFA-CT and the VFA-BIA

The measurement error between the VFAs was estimated using the two methods. The measurement error was defined as VFA-CT − VFA-BIA, and % measurement error was defined as {(VFA-CT − VFA-BIA)/VFA-CT)} × 100.

**Fig. 1:**
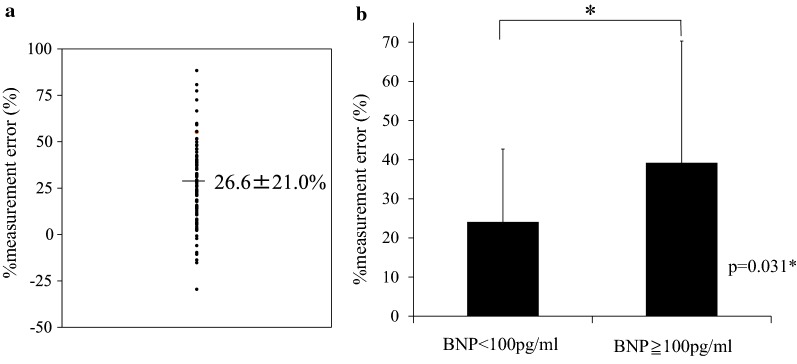
The measurement error between VFA by CT and dual BIA among patients with type 2 diabetes. The measurement of the VFA both by CT and by dual BIA was performed on the same day. The VFA by dual BIA was estimated after an overnight fast and urination. Then, before lunch, CT was performed, and the VFA at the umbilical level was determined by two independent researchers using the image analysis software. The measurement error between the two methods was expressed as % measurement error, which was calculated as follows: % measurement error = {(VFA-CT − VFA-BIA)/VFA-CT} × 100(%). **a** The distribution of % measurement error. **b** The % measurement error among different levels of BNP. Patients were divided into two groups according to their levels of BNP (cutoff value: 100 pg/mL). Data are expressed as mean ± standard deviation. *P < 0.05

**Fig. 2:**
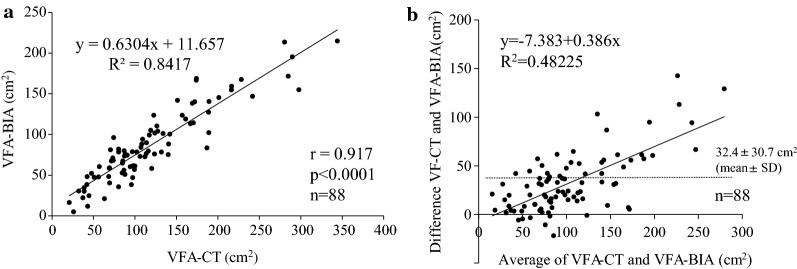
**a** The correlation between the VFA-CT and VFA-BIA. The correlation between the two methods was determined using Pearson’s correlation coefficient. Statistical significance was defined as P < 0.05. **b** Bland–Altman plots for comparison between VFA-CT and VFA-BIA

The mean % measurement error between the two methods was 26.6% ± 21.0% (Fig. 1a). We assessed correlation between % measurement error and variable factors such as age, gender, BMI, diabetes duration, HbA1c, fasting blood sugar (FBS), eGFR, BNP and history of heart failure (Table 2). In multivariate regression analysis, only BNP was independently correlated with % measurement error (Table 2). The % measurement error was higher among patients with BNP ≥ 100 pg/mL than among those with BNP < 100 pg/mL (39.2% ± 31.1% vs. 24.1% ± 18.6%; P = 0.03; Fig. 1b). After excluding patients with BNP ≥ 100 pg/mL, the VFA-BIA was significantly correlated with the VFA-CT (r = 0.917; P < 0.0001; Fig. 2a). In the patients with BNP ≥ 100 pg/mL the VFA-BIA was significantly but less correlated with VFA-CT (r = 0.749, P = 0.013*; Additional file 1: Figure S1). Bland–Altman plots was conducted to compare between VFA-CT and VFA-BIA. Mean difference in VFA-CT and VFA-BIA was 32.4 ± 30.7 cm^2^. Mean difference increased significantly as VFA-CT increased (Fig. 2b).

**Table 2:** The association between % measurement error and variables

	% measurement error (%)	
	β	P
Age	0.176	0.199
Sex	− 0.082	0.469
BMI	− 0.018	0.881
Diabetes duration	− 0.005	0.969
HbA1c	0.073	0.562
BS	− 0.115	0.340
eGFR	0.045	0.735
BNP	0.368	0.003*
History of heart failure	0.141	0.248

β: regression coefficient

*P < 0.05

### ROC analysis for identifying comorbid of hypertension and/or dyslipidemia with T2D

**Fig. 3:**
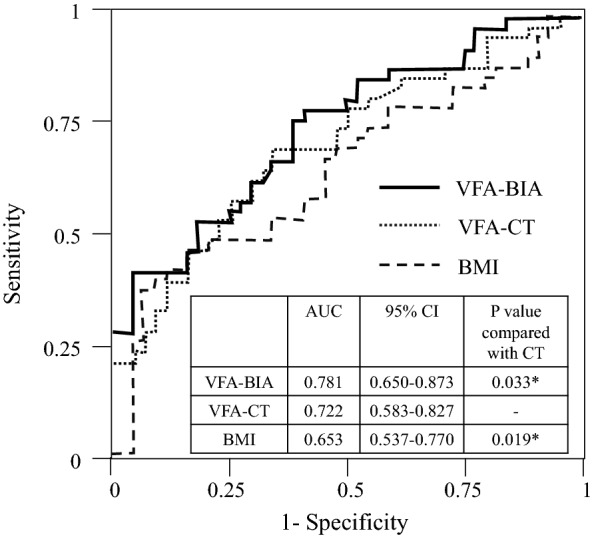
The ROC for identifying the presence of comorbid obesity-related cardiovascular risk factors. Cardiovascular risk factors were defined as hypertension (SBP ≥ 140 mmHg and/or DBP ≥ 90 mmHg or under treatment) and dyslipidemia (HDL-C < 40 mg/dL and/or triglycerides ≥ 150 mg/dL or under treatment) in addition to T2D. The curves are for the VFA-BIA (bold line), the VFA-CT (dotted line), and BMI (broken line)

We estimated the ROC of the VFA determined by the two methods for identifying the presence of obesity-related cardiovascular risk factors (hypertension and/or dyslipidemia) in patients with T2D. The respective optimal cutoff values for both factors were 99.4 cm^2^ for the VFA-CT (sensitivity, 73.8%; specificity, 65.2%), and 114.4 cm^2^ for the VFA-BIA (sensitivity, 45.2%; specificity, 95.6%). The AUC values were 0.653 [95% confidence interval (CI) 0.537–0.770] for the BMI, 0.722 (95% CI 0.583–0.827) for the VFA-CT, and 0.781 (95% CI 0.650–0.873) for the VFA-BIA. Importantly, the AUC in the ROC analysis for the VFA-BIA to detect the presence of comorbid cardiovascular risk factors was almost same as that for the VFA-CT (P = 0.62; Fig. 3). When only male patients were analyzed, the optimal cutoff values were 131.9 cm^2^ for the VFA-CT (sensitivity, 77.4%; specificity, 60.0%), and 114.4 cm^2^ for the VFA-BIA (sensitivity, 95.5%; specificity, 54.3%). The AUC values were 0.673 (95% CI 0.530–0.789) for the BMI, 0.742 (95% CI 0.609–0.842) for the VFA-CT, and 0.767 (95% CI 0.637–0.861) for the VFA-BIA. There was no significant difference in the AUC value between the VFA-BIA and VFA-CT (P = 0.397; Additional file 2: Figure S2).

The respective optimal cutoff values for co-existing hypertension or dyslipidemia alone were 88.4 cm^2^ and 107.3 cm^2^ for the VFA-CT, and 89.8 cm^2^ and 73.6 cm^2^ for the VFA-BIA. The AUC values for detecting hypertension alone were 0.606 [95% confidence interval (CI) 0.485–0.716] for the BMI, 0.680 (95% CI 0.548–0.788) for the VFA-CT, and 0.704 (95% CI 0.583–0.802) for the VFA-BIA (Additional file 3: Figure S3A), while the AUC values for detecting dyslipidemia alone were 0.616 [95% confidence interval (CI) 0.483–0.734] for the BMI, 0.666 (95% CI 0.544–0.769) for the VFA-CT, and 0.663 (95% CI 0.538–0.0.769) for the VFA-BIA (Additional file 3: Figure S3B). There was no significant difference in the AUC value between the VFA-BIA and VFA-CT in both analyses.

## Discussion

This study estimated the VFA by dual BIA and CT in patients with T2D. The VFA-BIA significantly correlated with the VFA-CT in patients with T2D without a potential subclinical heart failure. In the ROC analysis for detecting comorbid hypertension and/or dyslipidemia with T2D, which are major comorbidities of visceral obesity, the AUC value for the VFA-BIA was comparable with that for the VFA-CT, suggesting that the VFA-BIA can be used as well as VFA-CT to evaluate visceral obesity.

Visceral fat accumulation is more associated with cardiovascular risks and diseases than whole body fat accumulation especially in East Asian population that is generally less obese than Western countries [7, 8]. The VFA estimated by CT has been reported as a good surrogate marker of obesity-related disorders, especially such as hypertension, dyslipidemia and glucose intolerance [9–12]. Visceral fat accumulation is positively associated with diabetes and cardiovascular diseases, when subcutaneous adipose tissue is not [22]. The patients with visceral fat accumulation had low muscle quality that is associated with more frequent cardiovascular disease [23]. These reports suggest the importance of measuring visceral fat accumulation.

In this study, we found that the measurement error between the VFAs estimated by the two methods was relatively higher among patients with BNP ≥ 100 pg/mL than among those with BNP < 100 pg/mL. Based on the several papers including NICE Guideline No. 5 issued by National Clinical Guideline Centre (UK), the cutoff value of BNP is 100 pg/mL for the diagnosis of potential heart failure [15–17]. Thus, patients in our study may have a potential fluid retention that could affect the bioimpedance. In fact, there are several reports mentioning that overhydration status influenced whole body bioimpedance in patients with hypertension, CKD, proteinuria and hemodialysis [24, 25]. In our study, the VFA was calculated with the following three variables by dual BIA: abdominal shape, fat-free volume (FFV), and subcutaneous fat volume (SFV). Precisely, the VFA was calculated as follows: (abdominal area) − FFV − SFV [13]. In the present study, the FFV was primarily determined by the impedance of the entire abdomen, which is decreased with highly conductive water retention. Conversely, the SFV was determined by the constant current of the abdominal surface, which was barely affected by the water retention. Hence, in our study, the VFA was underestimated in patients with fluid retention, possibly explaining why patients with higher levels of BNP had a smaller VFA estimated by dual BIA. Hence, we excluded patients with BNP ≥ 100 pg/mL and performed further analyses. After excluding such patients, the VFA-BIA correlated significantly with the VFA-CT, exhibiting a high correlation coefficient of 0.917. These results indicated the clinical usefulness of dual BIA in patients with T2D without potential heart failure.

To the best of our knowledge, this is the first study to examine the correlation between the VFA-CT and VFA-BIA in patients with T2D. In this study, performing dual BIA and CT on the same day increased the robustness of our findings. In previous studies about general population, VFA estimated by impedance analysis correlated with VFA estimated by CT [26–30] and also correlated with parameters of obesity such as BMI, waist circumference and waist-hip ratio [31]. Gomez et al. and Park et al. reported bioimpedance analysis tends to underestimate VFA compared with CT [27–30]. Difference between VFA estimated by CT and BIA was large especially in the subjects with high BMI and large VFA estimated by CT [29, 30]. Our data with T2D patients are compatible to these previous studies. The reason why there is the proportional bias between the two methods in the morbid obese people is unknown. To compare the clinical usefulness of the two methods in those people, further studies such as examining the association of VFAs with cardiovascular hard events are necessary.

In the ROC analysis for detecting the comorbid hypertension and/or dyslipidemia, the VFA-BIA detected comorbid risk factors better than BMI. The AUC of the ROC analysis exhibited no significant differences between dual BIA and CT, although the AUC of the VFA-BIA tended to be larger than that of the VFA-CT. Several studies have demonstrated the utility of VFA-CT in detecting hypertension, dyslipidemia, and glucose intolerance [9–12]. Our results indicated that the VFA-BIA is clinically useful as a noninvasive and inexpensive substitute for the VFA-CT.

There are some limitations in this study. We enrolled relatively few patients with high prevalence of hypertension and dyslipidemia. We used single-measurement blood pressure data for the definition of hypertension, which can limit the result. However, 92% of the hypertensive patients were defined by receiving antihypertensive drugs in this study. Thus, we believe the effect of the single measurement was relatively low. We didn’t examine correlation of VFA-BIA to occurrence of cardiovascular diseases. To overcome these limitations, further study with more patients is necessary.

## Conclusion

In conclusion, the present study demonstrated that in patients with T2D without elevated BNP > 100 pg/mL as indicator for fluid accumulation interfering with BIA, estimation of visceral fat accumulation by dual BIA significantly correlates with that by CT. The VFA by dual BIA can also evaluate visceral obesity as it detects comorbid cardiovascular risk factors similar to the VFA by CT. Dual BIA is noninvasive and cost-effective as compared with CT and can thus be used as an alternative to CT as a standard method for estimating the VFA in patients with diabetes.

## Supplementary information

**Additional file 1: Figure S1.** The correlation between the VFA-CT and VFA-BIA among the patients with BNP > 100 pg/mL.

**Additional file 2: Figure S2.** The ROC for identifying the presence of comorbid obesity-related cardiovascular risk factors among male patients.

**Additional file 3: Figure S3. A** The ROC for identifying the presence of hypertension. **B** The ROC for identifying the presence of dyslipidemia.

## Data Availability

The datasets generated and/or analyzed during the current study are not publicly available because the informed consent did not cover the publication of the raw data. However, these are available from the corresponding author only if they are used for the validation of the paper.

## Abbreviations

T2D: type 2 diabetes
CT: computed tomography
BIA: bioelectrical impedance analysis
VFA: visceral fat area
ROC: receiving operating characteristic
AUC: area under the curve
BNP: brain natriuretic peptide
eGFR: estimated glomerular filtration rate
WC: waist circumference
BMI: body mass index
WHR: waist-to-hip ratio
HDL-C: high-density lipoprotein cholesterol
SBP: systolic blood pressure
DBP: diastolic blood pressure
HOMA-IR: a homeostasis model assessment for insulin resistance
FFV: free-fat volume
SFV: subcutaneous fat volume
