# Supplementary material for: Efficacy of visceral fat estimation by dual bioelectrical impedance analysis in detecting cardiovascular risk factors in patients with type 2 diabetes
Source: Cardiovasc Diabetol. 2019 Oct 22;18:137. doi: 10.1186/s12933-019-0941-y (PMC6805489; doi:10.1186/s12933-019-0941-y)
Supplement: Supplementary file 1 — Additional file 1: Figure S1. The correlation between the VFA-CT and VFA-BIA among the patients with BNP > 100 pg/mL. [file 12933_2019_941_MOESM1_ESM.pdf]

Figure.S1

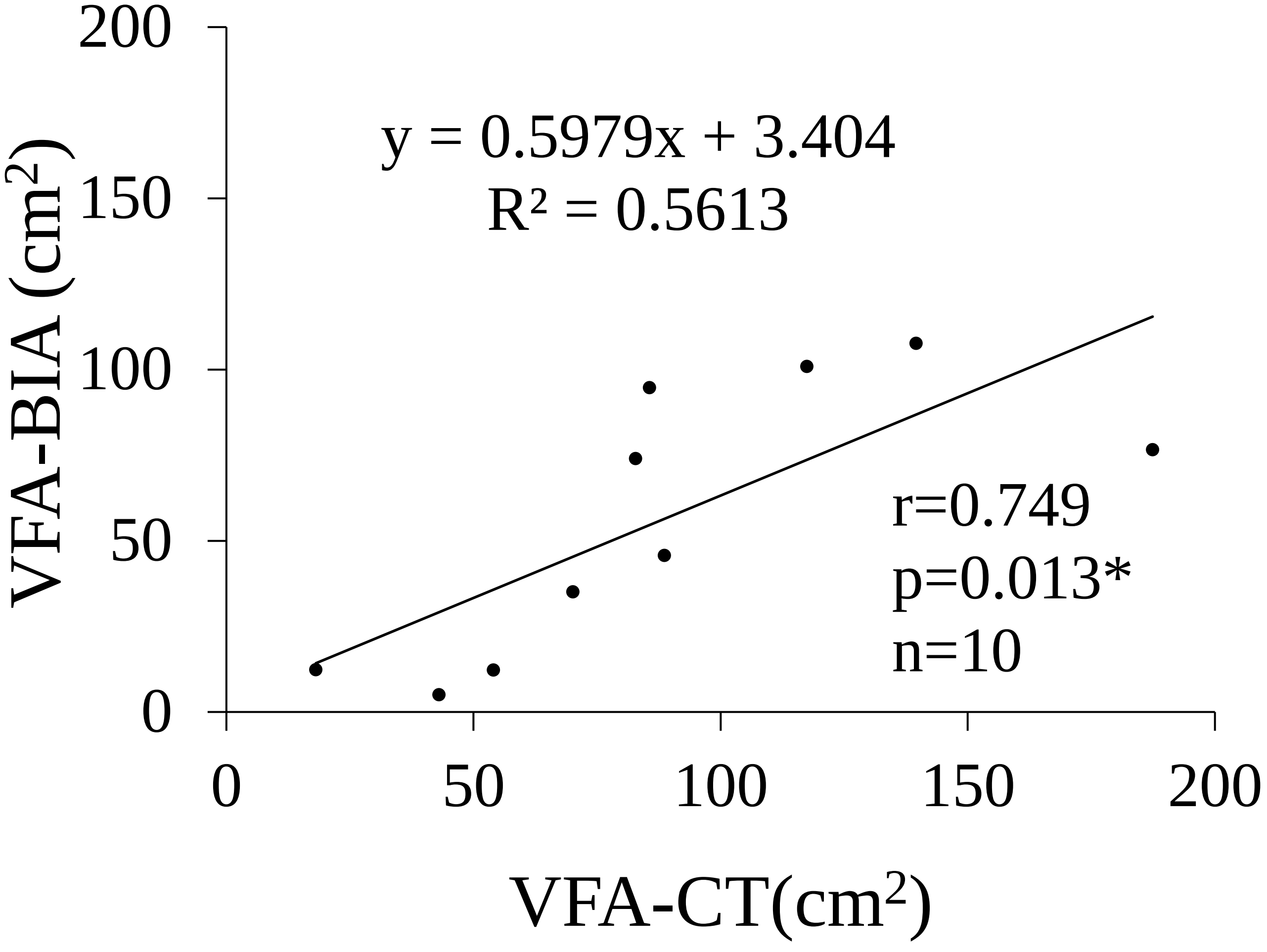

Figure.S1 The correlation between the VFA-CT and VFA-BIA among the patients with BNP> 100 pg/mL. The correlation between the two methods was determined using Pearson's correlation coefficient. Statistical significance was defined as $P < 0.05$.
