# Supplementary material for: Efficacy of visceral fat estimation by dual bioelectrical impedance analysis in detecting cardiovascular risk factors in patients with type 2 diabetes
Source: Cardiovasc Diabetol. 2019 Oct 22;18:137. doi: 10.1186/s12933-019-0941-y (PMC6805489; doi:10.1186/s12933-019-0941-y)
Supplement: Supplementary file 2 — Additional file 2: Figure S2. The ROC for identifying the presence of comorbid obesity-related cardiovascular risk factors among male patients. [file 12933_2019_941_MOESM2_ESM.pdf]

Figure. S2

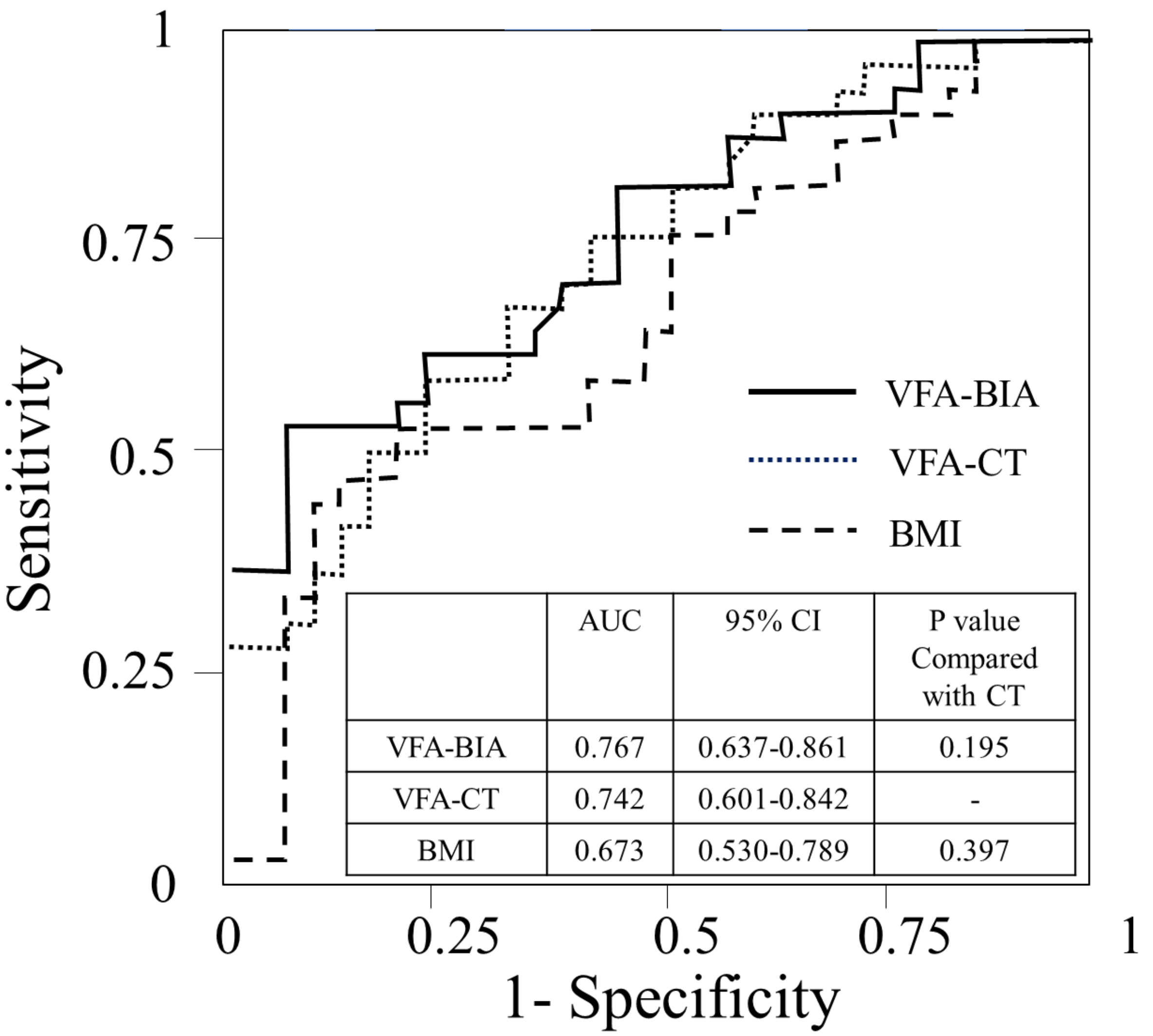

	AUC	95% CI	P value Compared with CT
VFA-BIA	0.767	0.637-0.861	0.195
VFA-CT	0.742	0.601-0.842	-
BMI	0.673	0.530-0.789	0.397

Figure.S2 The ROC for identifying the presence of comorbid obesity-related cardiovascular risk factors among male patients. Cardiovascular risk factors were defined as hypertension (SBP ≥ 140 mmHg and/or DBP ≥ 90 mmHg or under treatment) and dyslipidemia (HDL-C < 40 mg/dL and/or triglycerides ≥ 150 mg/dL or under treatment) in addition to T2D. The curves are for the VFA -BIA (bold line), the VFA-CT (dotted line), and BMI (broken line).
