# Supplementary material for: Efficacy of visceral fat estimation by dual bioelectrical impedance analysis in detecting cardiovascular risk factors in patients with type 2 diabetes
Source: Cardiovasc Diabetol. 2019 Oct 22;18:137. doi: 10.1186/s12933-019-0941-y (PMC6805489; doi:10.1186/s12933-019-0941-y)
Supplement: Supplementary file 3 — Additional file 3: Figure S3. A The ROC for identifying the presence of hypertension. B The ROC for identifying the presence of dyslipidemia. [file 12933_2019_941_MOESM3_ESM.pdf]

Figure.S3A

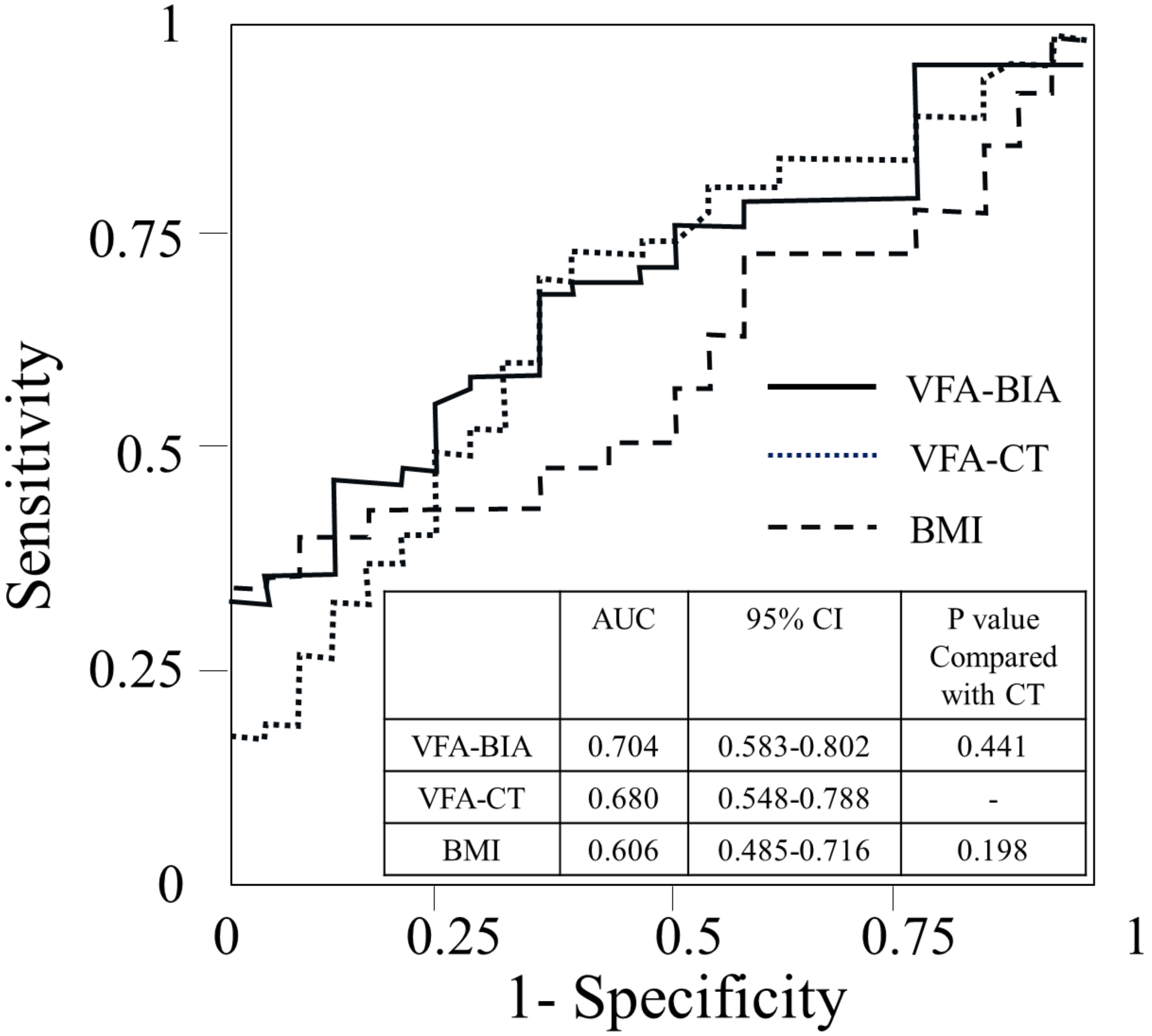

	AUC	95% CI	P value Compared with CT
VFA-BIA	0.704	0.583-0.802	0.441
VFA-CT	0.680	0.548-0.788	-
BMI	0.606	0.485-0.716	0.198

Figure.S3A The ROC for identifying the presence of hypertension. Hypertension was defined as SBP ≥ 140 mmHg and/or DBP ≥ 90 mmHg or under treatment. The curves are for the VFA -BIA (bold line), the VFA-CT (dotted line), and BMI (broken line).

Figure.S3B

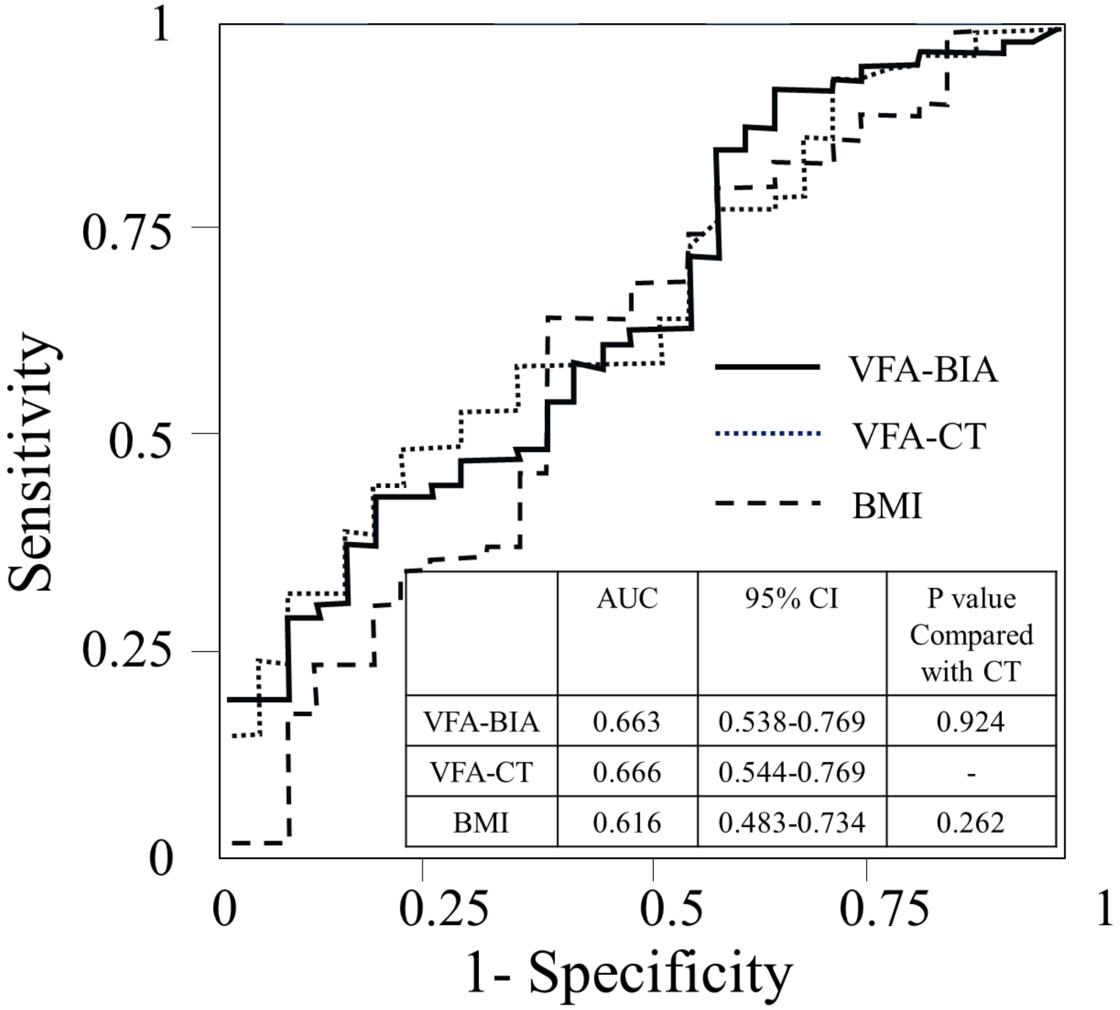

	AUC	95% CI	P value Compared with CT
VFA-BIA	0.663	0.538-0.769	0.924
VFA-CT	0.666	0.544-0.769	-
BMI	0.616	0.483-0.734	0.262

Figure.S3B The ROC for identifying the presence of dyslipidemia. Dyslipidemia was defined HDL-C < 40 mg/dL and/or triglycerides ≥ 150 mg/dL or under treatment. The curves are for the VFA -BIA (bold line), the VFA-CT (dotted line), and BMI (broken line).
